# Supplementary material for: Heat denaturation enables multicolor X10-STED microscopy
Source: Sci Rep. 2023 Apr 1;13:5366. doi: 10.1038/s41598-023-32524-5 (PMC10067834; doi:10.1038/s41598-023-32524-5)
Supplement: Supplementary file 1 — Supplementary Information. [file 41598_2023_32524_MOESM1_ESM.pdf]

Supporting information

Heat denaturation enables multicolor X10-STED microscopy

Saal, K.-A.[1,*]; Shaib, A.H.[1]; Mougios, N.[1,3]; Crzan, D.[1]; Opazo, F. [1,2,3]; Rizzoli, S.O.[1,3,*]

[1] University Medical Center Göttingen, Department of Sensory- and Neurophysiology, Humboldtallee 23, 37073 Göttingen, Germany

[2] NanoTag Biotechnologies GmbH, Rudolf Wissell Str. 28a, Rudolf-Wissell-Str. 28a 37079 Göttingen, Germany

[3] Center for Biostructural Imaging of Neurodegeneration (BIN), Von-Sieboldt-Str. 3a, 37075 Göttingen, Germany.

***** Correspondence: kim-ann.saal@uni-goettingen.de, Tel.: (+0049 551 3969675); srizzol@gwdg.de, Tel.: (+0049 551 395911)

Table of content

Supplementary tables

- **Table 1:** Specimen thickness and resulting expansion factor in X10ht

Supplementary Figures

- **Figure S1:** Optimization of autoclaving temperature.

- **Figure S2:** Comparison of expansion factor after the enzymatic digestion and autoclaving methods.

- **Figure S3:** STED imaging of X10ht expanded U2OS cells.

- **Figure S4:** Application of the X10ht protocol applied to the investigation of Nup96 in U2OS-mEGFP cells.

- **Figure S5:** Exemplary confocal images show the application of different signal amplification systems in primary neuronal cultures, using different fluorophores.

- **Figure S6:** Analysis of 3-color STED images to identify isolated synaptic vesicles in X10ht.

- **Figure S7:** Confocal and STED RAW images of synaptic vesicles shown in main Figure 6 including photon count colormaps

- **Figure S8:** Decorrelation analysis to obtain an alternative resolution estimation for X10ht-STED imaging.

- **Figure S9:** Comparison of resolution of VGLUT1-BT-AS635P in not expanded and X10ht processed neuronal cultures.

- **Figure S10:** Decorrelation analysis of VGLUT1 stained before the X10ht expansion procedure, or after the autoclaving (AC) step.

Supplementary tables

Table 1: Specimen thickness and resulting expansion factor in X10ht

Figure	specimen	thickness	Expansion Factor
Figure 1	U2OS Cell line	~ 10-20 µm	10
Figure 2	Neuronal culture	~ 10-20 µm	10
Figure 3	Brain slice	150 µm	6
Figure 4	Neuronal culture	~ 10-20 µm	10
Figure 5	Neuronal culture	~ 10-20 µm	10
Figure 6	Synaptic vesicle monolayer	~ 30-40 nm	10
Supplementary Figure S1	Baby Hamster kidney cell line	~ 10-20 µm	10
Supplementary Figure S2	U2OS Cell line	~ 10-20 µm	10
Supplementary Figure S3	U2OS Cell line	~ 10-20 µm	10
Supplementary Figure S4	U2OS Cell line	~ 10-20 µm	10
Supplementary Figure S5	Neuronal culture	~ 10-20 µm	10
Supplementary Figure S6	Synaptic vesicle monolayer	~ 30-40 nm	10
Supplementary Figure S7	Synaptic vesicle monolayer	~ 30-40 nm	10
Supplementary Figure S8	Synaptic vesicle monolayer	~ 30-40 nm	10
Supplementary Figure S9	Neuronal culture	~ 10-20 µm	10
Supplementary Figure S10	Neuronal culture	~ 10-20 µm	10

Table S1: *This table describes the specimens used for X10ht, depicting the thickness and the resulting expansion factor.*

Supplementary figures

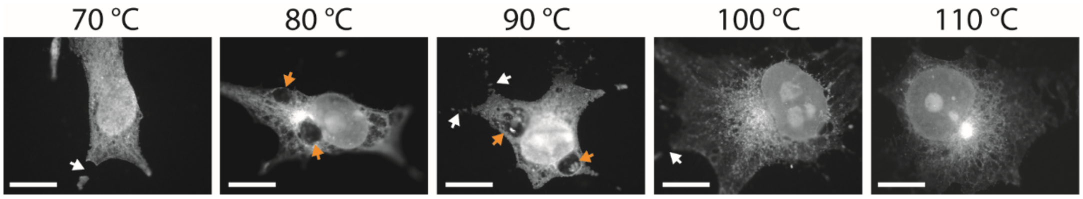

Figure S1: Optimization of autoclaving temperature. Exemplary epifluorescence images of expanded BHK cells are shown, treated with ascending temperatures from 70 – 110 °C during autoclaving. Lower temperatures resulted in cracked cells (white arrows), large cytosolic swellings (orange arrows), while higher temperature ensured the integrity of the cells and a higher expansion size. All cells are stained with NHS-Atto590 after autoclaving. Scale bar 100 µm, without taking the expansion factor into consideration.

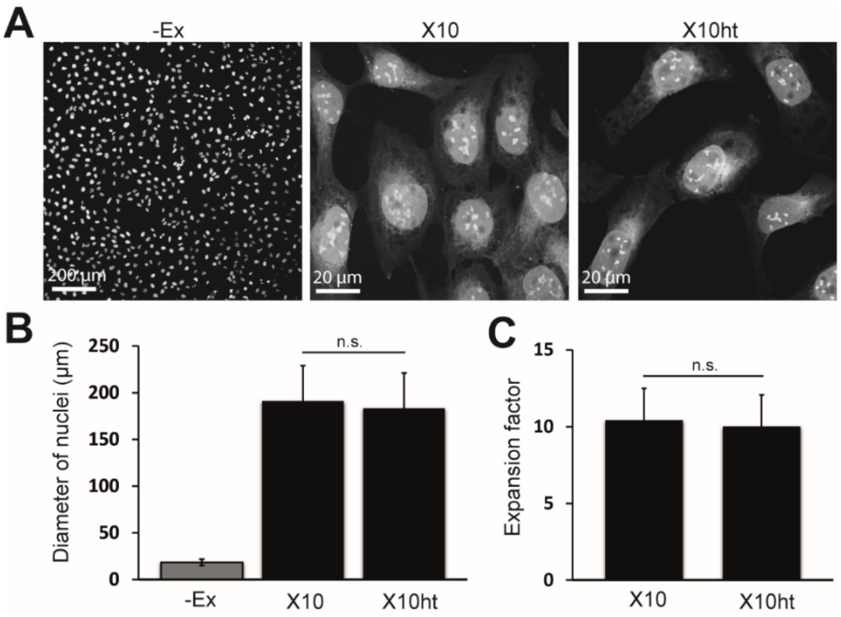

Figure S2: Comparison of expansion factor after the enzymatic digestion and autoclaving methods. A) Exemplary epifluorescence images for not expanded U2OS cells (-Ex) and after expansion with enzymatic homogenization with proteinase K (X10) or autoclaving at 110 °C (X10ht). All cells are stained with NHS-AF546 after homogenization. **B)** The analysis of the nucleus size revealed no significant (n.s.) difference in the size of expansion between the two protocols, and an expansion factor of ~10 for both conditions (shown in **C**). N = 29 analyzed cells for –Ex, 74 analyzed cells for X10 and 49 analyzed cells for X10ht obtained from two independent experiments.

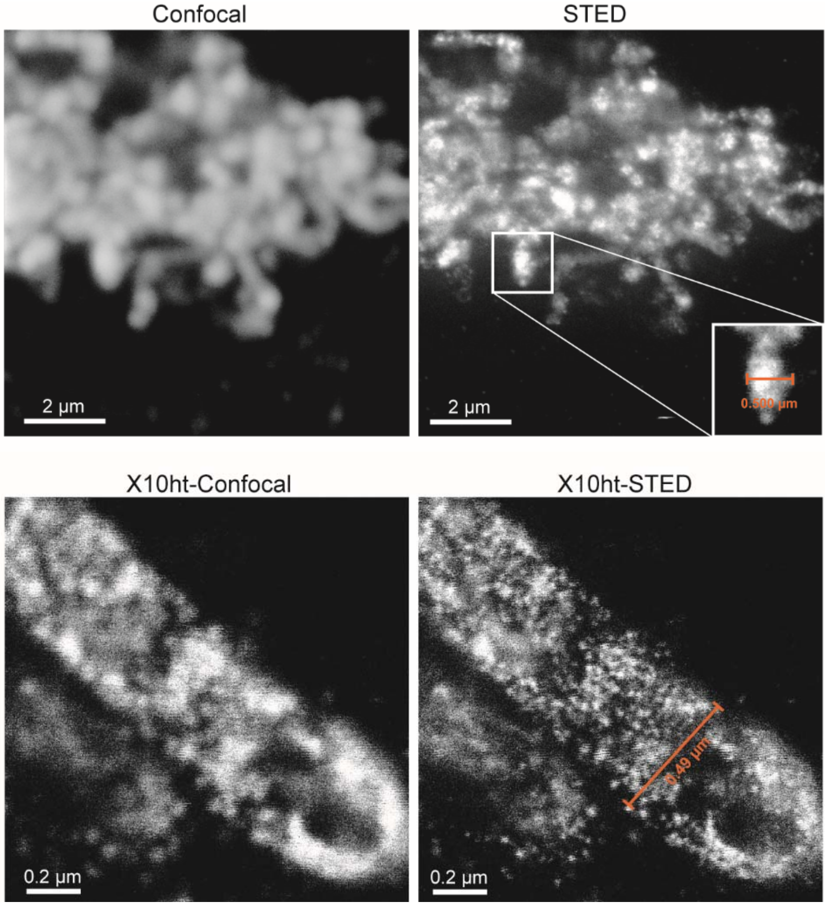

Figure S3: STED imaging of X10ht expanded U2OS cells. In the upper row, mitochondria imaged with STED show their typical tubular shape, with diameters of around 350-500 nm. This appearance is fully retained after X10ht expansion, and the measurement of the mitochondrial diameter confirms the ten-fold expansion of these organelles.

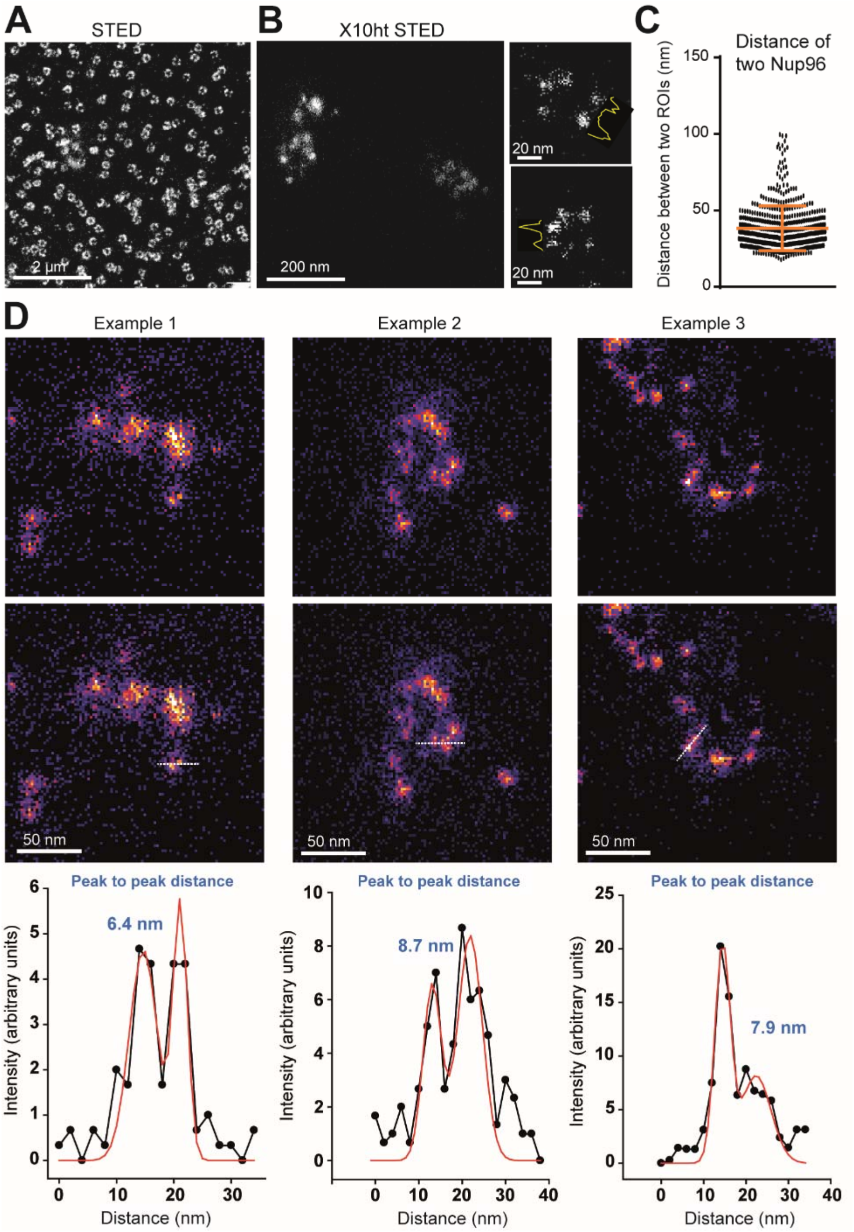

Figure S4: Application of the 10Xht protocol applied to the investigation of Nup96 in U2OS-mEGFP cells. A) STED image of not expanded Nup96 (STED). **B)** Nup96 ring-like structures after X10ht, imaged in STED. Additional examples are shown in the smaller panels, with the line scans in yellow depicting the profiles of two adjacent corners of a NPC. **C)** Quantification of the distance between the corners (mean value ~38 nm). N = 516 Nup96 subunits from 58 images from one experiment were analyzed. **D)** Three examples for the analysis of the distance between two neighbored spots. It is possible that spots found within a few nm of each other represent multiple fluorophores linked to the same Nup96 subunit, before expansion.

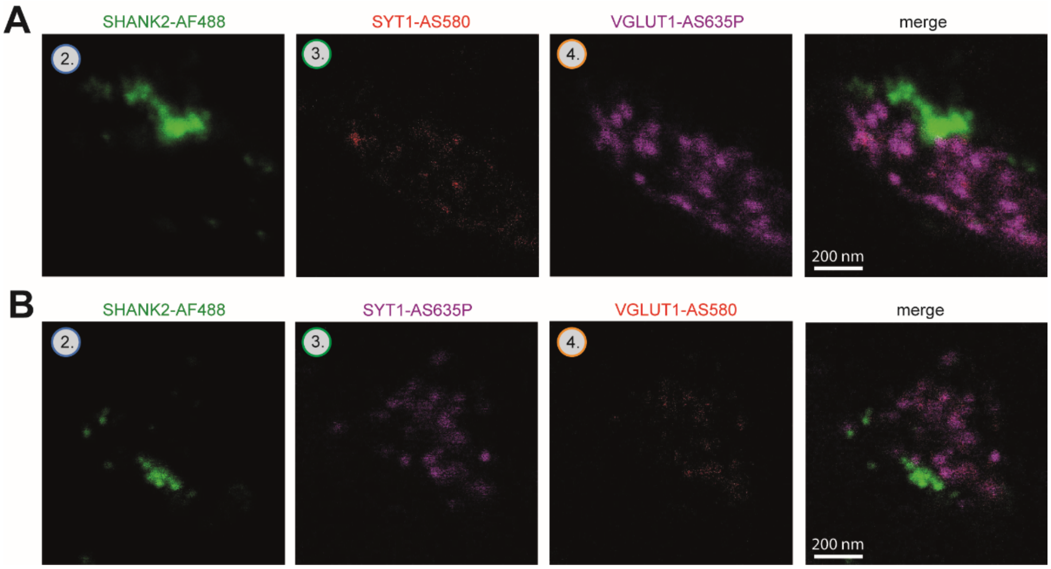

Figure S5: Exemplary confocal images show the application of different signal amplification systems in primary neuronal cultures, using different fluorophores. A) The combination of the AF488 system was used for labeling postsynaptic compartments (SHANK2) in green, together with NbSYT1-BT + AbAS580 and the NbALFA-SpaMo amplification to depict VGLUT1 with AbAS635P. **B)** The same immunostaining with a different combination of fluorophores for SYT1 and VGLUT1.

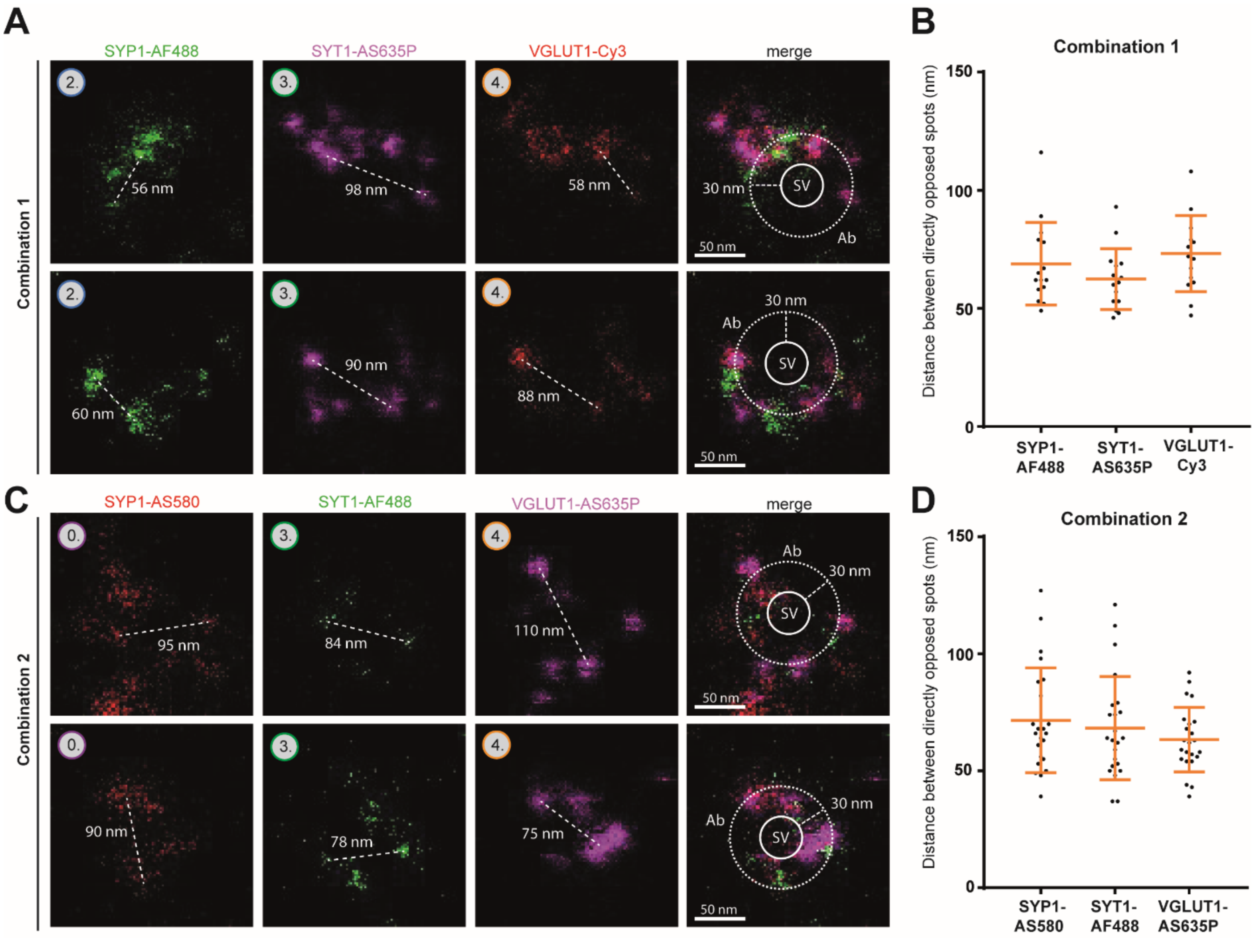

Figure S6: Analysis of 3-color STED images to identify isolated synaptic vesicles in X10ht. A) Two representative examples of the vesicle proteins SYP1, SYT1 and VGLUT1, immunostained with different amplification systems and fluorophore combinations. **B)** An analysis of distances between the spots (white dashed lines in **A)** of each channel. N = 15 images with several spot distances were analyzed. **C)** The same analysis, for a different fluorophore combination, with a similar quantification in panel **D)** reproduced the results of B. N = 21 images with several spot distances were analyzed. In the merged pictures, the synaptic vesicle (SV) is depicted by the inner circle (continuous line) and the region where the antibodies (Ab) are probably located is illustrated as the larger dashed circle. Data are given as individual data points, mean ± SD.

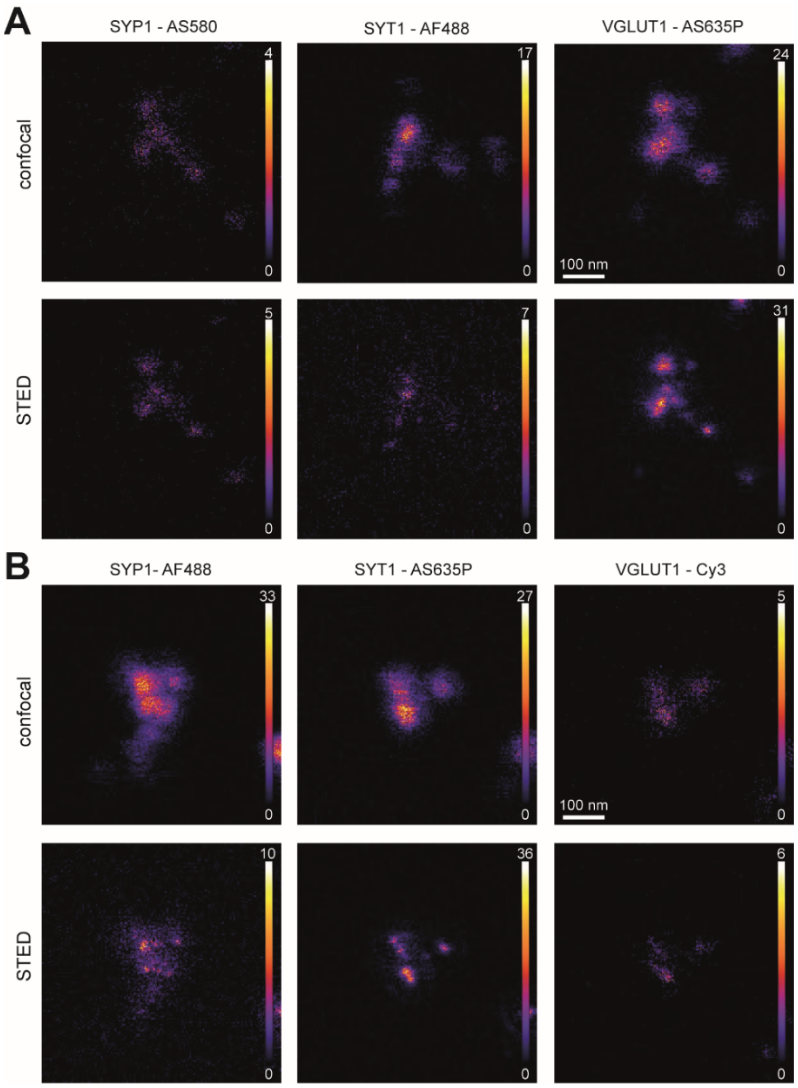

Figure S7: Confocal and STED RAW images of synaptic vesicles shown in the main Figure 6, as photon count colormaps. A) Exemplary staining combination for SYP1, SYT1 and VGLUT1, revealed by AS580, AF488 and AS635P, respectively. **B)** Same presynaptic targets with another selection of fluorophores. Low photon counts for AS580 and Cy3 result from a low max. laser excitation power in the used system.

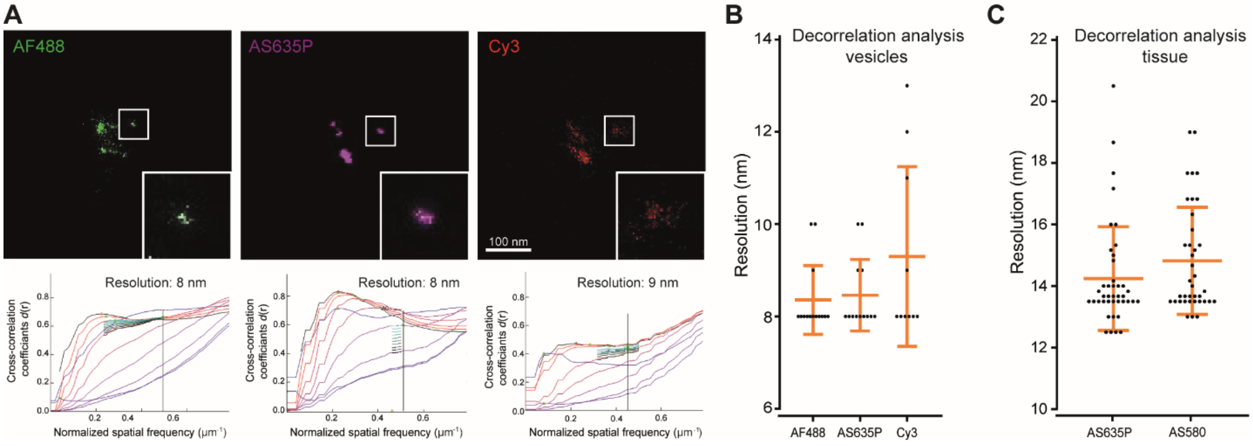

Figure S8: Decorrelation analysis to obtain an alternative resolution estimation for X10ht-STED imaging. A) Representative images of immunostainings of different synaptic vesicle proteins and their corresponding decorrelation analysis after Descloux and colleagues [37] in the framed region of interest. The colored lines are all high-pass filtered decorrelation functions; blue to black lines are the decorrelation functions with refined mask radius and highpass filtering range. The vertical line represents the cut-off kc. **B)** Quantitative analysis of the resolution of different spots. N = 14 images analyzed for AF488, 13 images analyzed for AS635P, and 11 images analyzed for Cy3 all from one experiment. **C)** Decorrelation analysis of fluorescent Bassoon-AS635P or Homer1-AS580 signals in 6-fold expanded 150 µm thick tissue slices, revealing resolutions between 12 and 20 nm. N = 41 ROI analyzed for AS635P and 38 ROI for AS580 generated from 26 images of 2 gels.

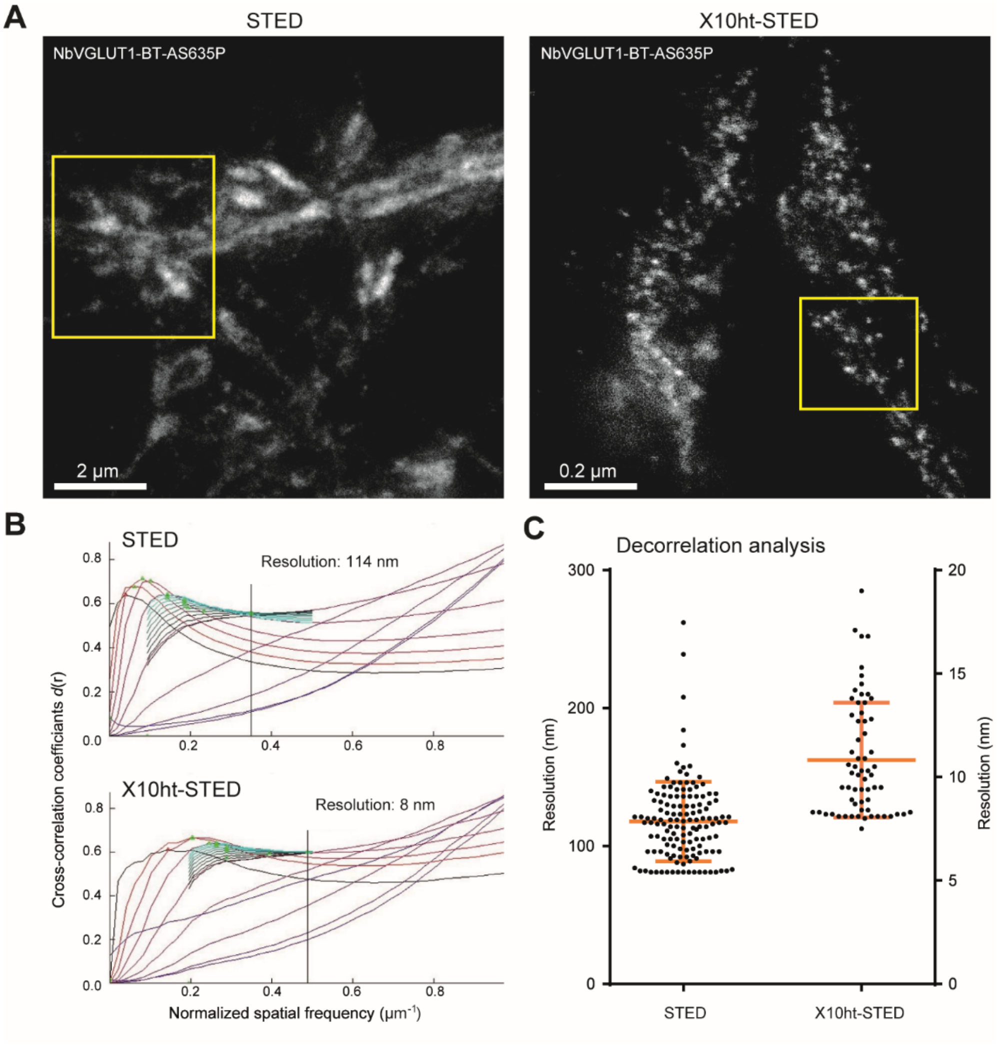

Figure S9: Comparison of resolution of VGLUT1-BT-AS635P in not expanded and X10ht processed neuronal cultures. **A)** shows representative STED images of primary hippocampal neurons stained with the biotin amplification system. Rectangles indicate the respective selected ROIs for decorrelation analysis, which is depicted in **B)**. **C)** Quantification of the resolution of VGLUT1-positive ROIs. N = 30 images with several analyzed ROIs out of 3 independent experiments for not expanded, and N = 13 images with several analyzed ROIs out of 2 independent experiments for X10ht treated samples. Data are presented as individual data points, mean ± SD.

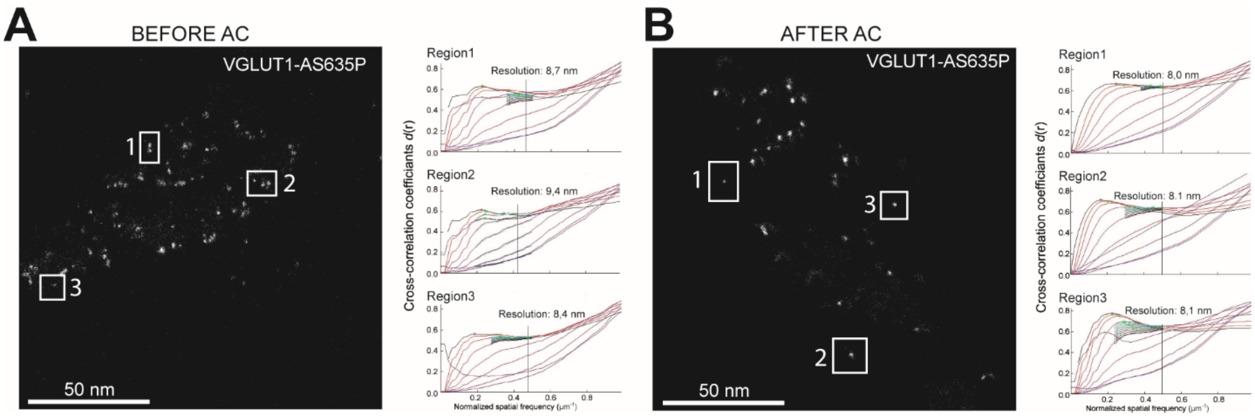

Figure S10: Decorrelation analysis of VGLUT1 stained before the X10ht expansion procedure, or after the autoclaving (AC) step. Exemplary regions of selection are marked as white rectangle in the STED images, generated from NbALFA.SpaMo labeling BEFORE AC (A) or from labeling with SpaMo AFTER AC B). The respective decorrelation curves show in both conditions resolutions between 8 – 10 nm.
